# Experiences and Perceptions of Older Adults with Lower-Risk Hormone Receptor-Positive Breast Cancer about Adjuvant Radiotherapy and Endocrine Therapy: A Patient Survey

**DOI:** 10.3390/curroncol28060436

**Published:** 2021-12-08

**Authors:** Marie-France Savard, Mashari Jemaan Alzahrani, Deanna Saunders, Lynn Chang, Angel Arnaout, Terry L. Ng, Muriel Brackstone, Lisa Vandermeer, Tina Hsu, Ari Ali Awan, Katherine Cole, Gail Larocque, Mark Clemons

**Affiliations:** 1 Department of Medicine, Division of Medical Oncology, The Ottawa Hospital and the University of Ottawa, Ottawa, ON K1H 8L6, Canada; mashari_220@hotmail.com (M.J.A.); teng@toh.ca (T.L.N.); thsu@toh.ca (T.H.); aawan@ohri.ca (A.A.A.); katcole@toh.ca (K.C.); mclemons@toh.ca (M.C.); 2 Cancer Therapeutics Program, Ottawa Hospital Research Institute, Ottawa, ON K1Y 4E9, Canada; dsaunders@ohri.ca (D.S.); anarnaout@toh.ca (A.A.); lvandermeer@ohri.ca (L.V.); 3 Department of Radiology, Division of Radiation Oncology, The Ottawa Hospital Cancer Centre and the University of Ottawa, Ottawa, ON K1H 8L6, Canada; lychang@toh.ca; 4 Department of Surgery, Division of General Surgery, The Ottawa Hospital and the University of Ottawa, Ottawa, ON K1H 8L6, Canada; 5 Division of Surgical Oncology, London Regional Cancer Program, London, ON N6A 4L6, Canada; muriel.brackstone@lhsc.on.ca; 6 The Ottawa Hospital, Ottawa, ON K1H 8L6, Canada; galarocque@toh.ca

**Keywords:** older adults, elderly, breast cancer, endocrine therapy, radiation therapy, adjuvant, perceptions

## Abstract

Older patients with lower-risk hormone receptor-positive (HR+) breast cancer are frequently offered both radiotherapy (RT) and endocrine therapy (ET) after breast-conserving surgery (BCS). A survey was performed to assess older patients’ experiences and perceptions regarding RT and ET, and participation interest in de-escalation trials. Of the 130 patients approached, 102 eligible patients completed the survey (response rate 78%). The median age of respondents was 74 (interquartile range 71–76). Most participants (71%, 72/102) received both RT and ET. Patients felt the role of RT and ET, respectively, was to: reduce ipsilateral tumor recurrence (91%, 90/99 and 62%, 61/99) and improve survival (56%, 55/99 and 49%, 49/99). More patients had significant concerns regarding ET (66%, 65/99) than RT (39%, 37/95). When asked which treatment had the most negative effect on their quality of life, the results showed: ET (35%, 25/72), RT (14%, 10/72) or both (8%, 6/72). Participants would rather receive RT (57%, 41/72) than ET (43%, 31/72). Forty-four percent (44/100) of respondents were either, “not comfortable” or “not interested” in participating in potential de-escalation trials. Although most of the adjuvant therapy de-escalation trials evaluate the omission of RT, de-escalation studies of ET are warranted and patient centered.

## 1. Introduction

Over 30% of new breast cancers (BCs) are diagnosed in adults aged ≥ 70, and this proportion is rising [1,2]. Older patients diagnosed with early-stage hormone-receptor positive (HR+) disease who undergo breast-conserving therapy (BCS) commonly receive both adjuvant radiation therapy (RT) and endocrine therapy (ET) [3]. However, this treatment approach is based on randomized controlled trials that either exclude or underrepresent patients aged ≥ 70 [4,5,6,7,8].

Older patients with cancer tend to have more comorbid conditions, polypharmacy, poorer function and performance status, which all have implications on tolerance and compliance with treatment. Moreover, the competing risk of death in older adults with lower-risk HR+ BC render the adjuvant therapy benefits lesser than initially reported [9,10,11].

Prospective [12,13], retrospective [14,15], systematic review and meta-analysis [16,17,18] and computer modeling studies [19] have demonstrated that it is safe to omit either RT or ET without affecting distant metastasis and survival outcomes for older adults with lower-risk HR+ BC. However, the randomized trials on adjuvant therapy de-escalation mainly evaluated the omission of RT [18]. However, this body of evidence has not changed broader clinical practice, as most patients continue to receive both treatment modalities [3,20,21]. Furthermore, it remains unclear whether the best de-escalation strategy is RT alone or ET alone [9,22].

We conducted a survey among patients aged ≥70 who were offered adjuvant RT and ET for a lower-risk HR+ BC to better understand their perceptions with respect to adjuvant RT/ET and their interest in participating in clinical studies evaluating treatment de-escalation. The results of this survey will inform the decision-making process, include the patient’s voice in current clinical practice and align our research objectives with the patient’s needs and priorities.

## 2. Materials and Methods

### 2.1. Study Population

The survey’s target population was patients ≥ 70 years of age who were diagnosed with early-stage (node negative) HR+ breast cancer treated with BCS, and who had been offered both RT and ET by their treating team. Of note, in our health region, all patients who had a BCS or a mastectomy for an invasive breast cancer would be referred to both a medical oncologist and a radiation oncologist to discuss adjuvant therapies. The original study plan was to accrue 200 participants in 3 months to ensure a broad perspective on treatment. Furthermore, in order to capture a wide range of experience on the treatment and decision-making process, patients could be at any point in the course of their breast cancer treatment or surveillance from the time that both adjuvant therapies were discussed. On 1 November 2020, an amendment was approved to the protocol to include patients treated with a mastectomy to better reflect our institutional practice and improve recruitment (Supplementary Materials). In addition, we felt that these patients could present some valuable input on their perceptions of adjuvant therapies. Patients had to be able to provide verbal consent and complete the survey in English. Patients were accrued in two cancer centers: The Ottawa Hospital Cancer Center (TOH), including the General Campus and the Irving Greenberg Family Cancer Center (Ottawa, ON, CA), and The London Health Sciences Center (London, ON, CA).

### 2.2. Study Outcomes

The aim of this survey was to provide insight on the experiences, perspectives and expectations of older patients with regards to adjuvant ET and RT for lower-risk HR+ breast cancer. Furthermore, this survey sought to understand patient interests in participating in clinical trials evaluating the de-escalation of ET.

### 2.3. Survey Development

The patient survey was developed by a multidisciplinary team with demonstrated expertise in oncology, methodology and survey design [23,24], including medical oncologists (MC, MFS, TH), a radiation oncologist (LC), a surgical oncologist (AA), a nurse practitioner (GL) and a medical oncology resident (KC). It was also reviewed by non-healthcare professionals (DS, LV). The first part of the survey gathered information regarding patient demographics (age at diagnosis), health conditions (comorbidities, prescribed medications and self-rated health) and breast cancer medical context (type of adjuvant therapy received and the point at which they were in their breast cancer treatment) (8 items). The subsequent section evaluated the patients’ understanding of the benefits and risks of ET and RT, as well as the expected benefit threshold at which they would accept treatment or not (10 items). Then, patients were asked to provide their views on participating in a theoretical de-escalation study looking at omitting ET (2 items). Finally, patients were asked to share their own experiences regarding ET and/or RT, including side effects and impact on quality of life, and to identify which of the two they would opt for given the choice (14 items) (the complete survey is available in the “Supplementary Materials” Section).

### 2.4. Survey Implementation

Patients meeting eligibility criteria were approached and recruited by members within their circle of care (e.g., medical oncologists, radiation oncologists, general practitioners in oncology, nursing staff) during their routine clinic visits. Once verbal permission was given, interested patients had the choice of receiving a hard copy or an electronic version of the information sheet and survey. The electronic version was sent by email with a link to the anonymous survey on Microsoft Forms (on the secure Ottawa Hospital SharePoint site). Reminders were not sent to patients. The clinical research study associates (DS, LV) assisted in contacting interested patients, collecting completed surveys and gathering data. In addition, patients discharged from the oncology clinic to their family physicians through the Wellness Beyond Cancer Program of The Ottawa Hospital Cancer Center were also contacted (DS, MFS). This survey was approved by the Ontario Cancer Research Ethics Board (OCREB).

### 2.5. Data Analysis

Data were reported using descriptive statistics and analyzed using Microsoft Excel. The answer choices were summarized with observed proportions.

## 3. Results

### 3.1. Patient Characteristics

Between 29 July 2020 and 3 March 2021, 130 patients were approached: 114 in Ottawa and 16 in London. Among these, 27 (21%) declined participation (Ottawa 26, London 1) and 1 was deemed not eligible afterwards due to their age at diagnosis. Of the eligible candidates, 87 and 15 completed surveys were obtained from Ottawa and London, respectively. Patient characteristics are presented at Table 1. Among the 102 eligible respondents, the median age at BC diagnosis was 74 (interquartile range (IQR) 71–76). Forty-five percent (46/102) were ≥75. Most participants rated their health status as “good” (66%, 66/100). Excluding ET, the median and average number of prescribed medications taken per day by participants was three (IQR 2–4.75). The most commonly reported health problems were hypertension (49%, 50/102) and dyslipidemia (38%, 39/102).

At the time that the survey was completed, 71% (72/102) were receiving or had received both RT and ET, 12% (12/102) were receiving or had received RT only and 9% (9/102) ET only.

### 3.2. RT: Patient Experiences

Of the 100 respondents who answered RT related questions: 10% (10/100) had declined RT, 7% (7/100) had not yet started RT, 1% (1/100) were receiving RT and 82% (82/100) had completed RT (Table 1). The most common RT regimen received was 5 days per week for 3 weeks (72%, 61/85). In the 82 patients who completed RT, the main side effects reported were: skin redness (67%, 55/82) and fatigue (49%, 40/82). In patients who had completed RT at least 3 months ago, the ongoing common side effects were: fatigue (33%, 25/75) and breast pain (17%, 13/75) (Supplementary Materials).

**Figure 1:**
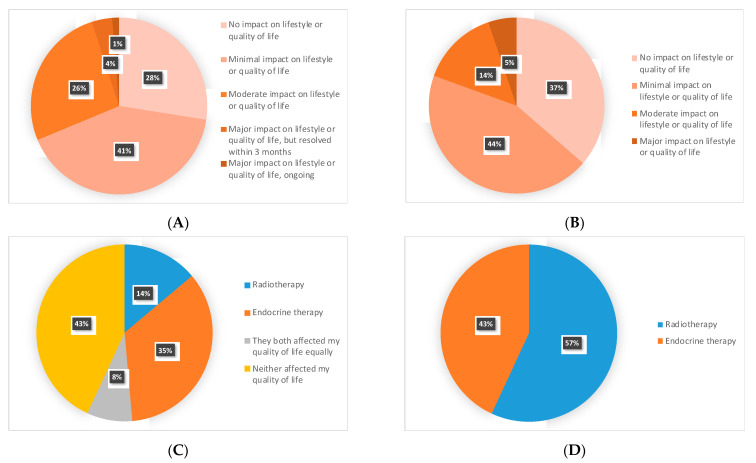
Radiotherapy versus endocrine therapy’s impact on quality of life and preferences: (**A**) impact of radiotherapy on quality of life/lifestyle; (**B**) impact of endocrine therapy on quality of life/lifestyle; (**C**) which treatment affects your quality of life the most?; (**D**) which treatment would you rather receive?

When participants were asked how RT affected their quality of life (QoL) or lifestyle, 26% (21/80) reported moderate impact, 4% (3/80) reported major impact that was resolved within 3 months and 1% (1/80) major ongoing impact. (Figure 1).

### 3.3. ET: Patient Experiences

Eighteen percent (18/101) declined ET, 7% (7/101) had not yet started ET, 65% (66/101) were taking ET and 7% (7/101) completed 5 years of ET. Two (2/101) reported that ET was not recommended to them (Table 1). Among participants who had either received or were still taking ET, 41% (33/80) took tamoxifen, 41% (33/80) anastrozole, 25% (20/80) letrozole and 6% (5/80) more than one type of ET. Eighty-three percent (66/80) reported that they were complying with the prescribed ET and 15% (12/80) did not take it at all. Among patients not taking ET, two completed 5 years of ET and the other ones had stopped taking it (13%, 10/80) (Supplementary Materials).

The main side effects reported for ET were hot flashes (45%, 36/80), joint pain (45%, 36/80) and fatigue (41%, 33/80) (Supplementary Materials). Regarding the impact of ET on QoL or lifestyle, 81% (62/77) reported no impact or minimal impact, 14% (11/77) reported moderate impact and 5% (4/77) reported major impact (Figure 1).

### 3.4. RT vs. ET: Impact on QoL and Patients’ Preferences

Among 72 respondents who had received both RT and ET, 43% (31/72) reported that neither RT nor ET affected their QoL, 35% (25/72) that ET affected their QoL more than RT, 14% (10/72) that RT affected their QoL more than ET, 8% (6/72) that RT and ET affected their QoL equally (Figure 1). When participants who received both RT and ET were asked which of RT or ET that they would rather receive, 57% (41/72) preferred RT over ET (Figure 1).

### 3.5. Patient’s Perceptions and Expectations of RT and ET Benefits

Ninety-one percent (90/99) answered that RT would help reduce ipsilateral breast recurrence vs. 62% (61/99) for ET (Table 2). Fifty-six percent (55/99) vs. 49% (49/99) thought that RT vs. ET would improve their survival, respectively. Forty-four percent (44/99) felt that RT would help reduce metastatic recurrence vs. 49% (49/99) for ET, whereas 25% (25/99) felt that RT would help reduce the occurrence of a contralateral BC vs. 53% (52/99) for ET. Interestingly, 12% (12/99) for RT and 18% (18/99) for ET answered that they did not know what the treatment does, but were told that it is important for them, and 29% (29/99) believed that ET might cause side effects without benefits.

Sixty-one percent (58/95) selected that they had no significant concerns for RT vs. 34% (34/99) for ET. Twenty-five percent (24/95) were concerned about possible side effects of RT, which was doubled at 51% (50/99) for ET. Only 18% (17/95) were concerned about the possible lack of benefit of RT compared to 26% (26/99) for ET. For ET, 30% (30/99) were concerned about the impact on QoL.

### 3.6. Clinical Scenario Evaluating Decision Making

More than half of the participants selected that they would agree to take RT or ET for any degree of benefit in terms of ipsilateral breast recurrence (RT: 66%, 65/98; ET: 51%, 51/100), metastatic recurrence (RT: N/A; ET: 54%, 52/100) and overall survival (RT: N/A; ET: 57%, 56/100) at 5 years. The second most common answer was an absolute benefit threshold of at least 50% for every category: ipsilateral breast recurrence (RT: 8%, 8/98; ET: 15%, 15/100), metastatic recurrence (RT: N/A; ET: 12%, 12/100) and overall survival (RT: N/A; ET: 18%, 18/100) at 5 years (Table 3).

### 3.7. Patients’ Attitudes towards Omitting RT or ET, and a Future De-Escalation Clinical Trial

**Figure 2:**
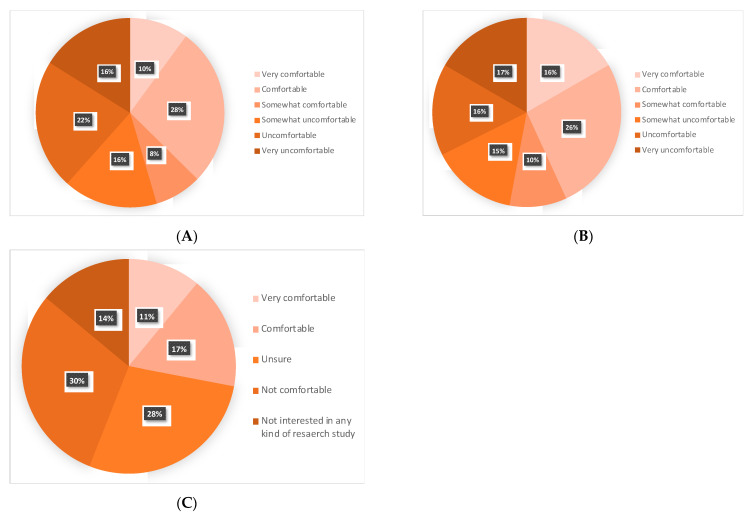
Attitudes toward omitting radiotherapy, endocrine therapy and participating in a de-escalation study: (**A**) omitting radiotherapy; (**B**) omitting endocrine therapy; (**C**) de-escalation study that omits endocrine therapy.

Participants were asked how comfortable they would be if their oncologists did not offer RT or ET based on their health and lower-risk cancer status (Figure 2): 45% (45/99) and 53% (54/102) would be comfortable to various degrees in omitting RT or ET, respectively.

Regarding their interest in participating in a hypothetical clinical trial that omits ET: 28% (28/100) would feel very comfortable or comfortable, 28% (28/100) unsure, 30% (30/100) not comfortable. Furthermore, 14% would not be interested in participating in any kind of research study (Figure 2).

## 4. Discussion

To our knowledge, this is the first survey on the perceptions of older adults with lower-risk HR+ BC towards RT and ET and de-escalation clinical trials. Although RT and ET are the two established adjuvant therapies for HR+ HER2- BC, evidence supporting their use in this population is based on extrapolation from studies that mainly accrued younger patients with higher risk BC. There is increasing interest in de-intensifying adjuvant therapies in older patients with biologically favorable BC defined as node-negative, small size, low-intermediate grade and HR+ BC [9]. To date, most of the prospective trials performed in older patients compared the omission of RT to standard of care, i.e., RT plus ET [25,26,27,28,29,30,31]. These studies demonstrated that while RT reduced the rate of ipsilateral breast tumor recurrence, neither the omission of RT nor ET impacts survival [14,17,18]. Limited data exist on ET omission [12,13,18] and clinical endpoints, such as QoL, safety and economics [9,18].

In this survey, almost half of the participants were aged 75 and older, providing a good representation of the elderly population. However, it is possible that highly educated and non-minority populations were over-represented, as we did not collect data on this. While many participants perceived themselves to be in good health, many reported comorbidities and most took several medications, as is common with aging. Notably, a substantial proportion of patients were taking four or more medications. For these patients, the addition of ET would result in polypharmacy [32], which can be associated with increased pill burden, increased likelihood of side effects and can have financial implications for patients. Thus, taking additional medication (ET) is not inconsequential for patients in this age category [33].

About 70% of the participants in this survey received or are planning to receive both RT and ET, which correlates with the most common adjuvant approach [3,20,21]. However, many appeared to misunderstand and/or overestimate the clinical benefits of RT and ET. For example, 44% believed that RT helps reduce metastatic recurrence and 25% felt it could reduce the risk of contralateral BC occurrence. Even though the survival benefit of RT and ET was not demonstrated [9,18,19], about 50% believed that it will improve their survival.

Surprisingly, while more than 50% would have taken RT/ET regardless of the magnitude of benefit, others would have taken RT/ET only if it yielded a level of efficacy that is much higher than what is demonstrated. One possible reason is that the concept of benefits relative to risks is difficult for physicians to explain and challenging for patients to grasp. This highlights a key aspect which must be accounted for and improved upon in order to help patients in their decision-making process. The relatively high proportion who would take adjuvant therapies regardless of the degree of benefit and the high ET compliance rate could also reflect the fact that a high proportion reported that treatments did not significantly impact their QoL, and/or could reflect a selection bias. These respondents may be fitter and are likely those who tolerated treatment. However, this remains an important finding, which points out that increased age does not always equate with a desire for less treatment or a shift in perception about what outcomes are important (e.g., overall survival vs. QoL), and emphasizes the importance of asking older patients about their values and preferences.

Participants preferred RT over ET in terms of side effects, expected benefits and QoL. Furthermore, a majority of patients treated with ET and RT would rather receive RT than ET and appeared to be more comfortable in omitting ET rather than RT. This may reflect technical and practical advances in RT, which have lessened late toxicities and reduced treatment numbers, rendering RT much more tolerable in recent years. This preference for RT over ET is also important, as it contrasts with the fact that almost all randomized controlled trials have focused on evaluating the omission of RT rather than ET [9], suggesting that studies evaluating the omission of ET are warranted. The necessity of such studies may be increasingly important with the adoption of 5-fraction RT courses, which were not standard at the time when participants received treatment [34] and may further skew patient preference towards ET omission.

Despite these findings and the interest from a medical perspective in minimizing harm due to over-treatment, up to 45% were not comfortable or did not want to participate in any kind of research study. A prior systematic review reported that 83% of the studies identified patient barriers as a major category that hindered the enrollment of older patients in clinical trials, of which 50% were due to patient concerns regarding the idea of experimentation [35]. This highlights the importance of both engaging patients during clinical study development and addressing their concerns to ensure that proposed studies are successful and inclusive of a wide range of older patients.

Our study has limitations. First, the COVID-19 pandemic directly affected our accrual. Fewer patients underwent surveillance mammography, and, therefore, a reduced number of new patients were seen. Furthermore, there were less opportunities for direct interactions as many patients were being seen virtually. In this survey, we included patients at different stages in their BC treatment journey to capture short- and long-term perspectives. This might be perceived as a limitation, since it makes the population less homogeneous and may introduce a recall bias if some were more than 5 years after their diagnosis. The survey only included patients who had been offered both ET and RT, and did not include patients who for whatever reason were not offered either modality. Furthermore, as most patients agreed to therapy, it is likely that these patients perceived a greater benefit to therapy and had a greater willingness to take therapy. Finally, this survey was completed without supervision and the treatments received could not be verified. In addition, some patients omitted answering certain questions. This might be due to inability to recall information, the length of the questionnaire or a lack of understanding.

## 5. Conclusions

Despite the paucity of data specifically addressing adjuvant therapy for older patients with lower-risk HR + BC, most participants surveyed received both RT and ET, and appeared to overestimate the clinical benefits of RT and ET. Older patients might be more interested in de-escalation clinical trials that omit ET, since they would rather receive RT than ET and were more comfortable in omitting ET. This patient survey will be a key instrument for including patient voices in the design of future clinical trials.

## Figures and Tables

**Table 1:** Patient demographics, health conditions and medical context.

	N	N (%)
Median Age (interquartile range)	74 (71–76)	
Age Group	102	
70–74		56 (55)
75–79		34 (33)
80+		12 (12)
Health status patient’s perception	100	
Excellent		20 (20)
Good		66 (66)
Fair		13 (13)
Poor		1 (1)
Bad		0 (0)
Number of prescribed medications/days	98	
0		10 (10)
1–3		52 (53)
4–5		23 (23)
6–9		10 (10)
10+		3 (3)
Health problems (past or current) *	102	
Diabetes		13 (13)
Hypertension		50 (49)
Dyslipidemia		39 (38)
Heart disease		7 (7)
Stroke		6 (6)
Kidney disease		4 (4)
Liver disease		2 (2)
Lung disease		15 (15)
Stomach ulcers		6 (6)
Thromboembolic disease		6 (6)
Other cancers		14 (14)
Memory problems		6 (6)
Mobility problems		18 (18)
Others		35 (34)
Type of adjuvant therapy received	102	
Radiotherapy alone		12 (12)
Hormonal therapy alone		9 (9)
Both		72 (71)
Radiotherapy progress status	100	
Declined		10 (10)
Planned in the future		7 (7)
Ongoing		1 (1)
Completed <3 months ago		7 (7)
Completed 3–6 months ago		11 (11)
Completed 6–12 months ago		27 (27)
Completed >12 months		37 (37)
Endocrine therapy progress status	101	
Declined		18 (18)
Planned in future		7 (7)
Started <3 months ago		8 (8)
Started 3–6 months ago		10 (10)
Started 6–12 months ago		17 (17)
Started >12 months ago		31 (31)
Completed 5 years		7 (7)
Others (not recommended, stopped for side effects)		3 (3)

* participants were able to choose more than one answer.

**Table 2:** Perception of radiotherapy and endocrine therapy.

Types of Benefits and Concerns	Radiotherapy		Endocrine Therapy	
	N	N(%)	N	N (%)
Benefits *	99		99	
Reduce ipsilateral tumor recurrence		90 (91)		61 (62)
Reduce occurrence of a contralateral breast cancer		25 (25)		52 (53)
Reduce metastatic recurrence		44 (44)		49 (49)
Survival benefit		55 (56)		49 (49)
Improvement in quality of life		35 (35)		12 (12)
Cause side effects without benefit		22 (22)		29 (29)
Don’t know		12 (12)		18 (18)
Others		1 (1)		5 (5)
Concerns *	95		99	
Possible side effects		24 (25)		50 (51)
Impact on quality of life		14 (15)		30 (30)
Impact on carrying daily activities		11 (12)		15 (15)
Lack of benefits		17 (18)		26 (26)
Treatment duration		5 (5)		11 (11)
Commuting for treatment		8 (8)		
No significant concerns		58 (61)		34 (34)
Others		2 (2)		1 (1)

* Participants were able to choose more than one answer; Boxes that are shaded grey indicated that question was not asked.

**Table 3:** Acceptable benefit threshold of radiotherapy and endocrine therapy.

Thresholds by Benefit Type	Radiotherapy		Endocrine Therapy	
	N	N(%)	N	N (%)
Ipsilateral breast recurrence at 5 years	98		100	
1%		0 (0)		3 (3)
5%		5 (5)		9 (9)
10%		6 (6)		6 (6)
15%		4 (4)		2 (2)
20%		6 (6)		9 (9)
30%		3 (3)		3 (3)
50%		8 (8)		15 (15)
Any possible benefit		65 (66)		51 (51)
Not important to me		1 (1)		2 (2)
Metastatic recurrence at 5 years			97	
1%				2 (2)
5%				10 (10)
10%				7 (7)
15%				2 (2)
20%				8 (8)
30%				2 (2)
50%				12 (12)
Any possible benefit				52 (54)
Not important to me				2 (2)
Survival at 5 years			99	
1%				0 (0)
2%				2 (2)
5%				7 (7)
10%				5 (5)
20%				7 (7)
30%				1 (1)
50%				18 (18)
Any possible benefit				56 (57)
Not important to me				3 (3)

Boxes that are shaded grey indicated that question was not asked.

## Data Availability

Data is available from the author upon request and with permission from the Ontario Cancer Research Ethics Board.

## Supplementary Materials

The following are available online at https://www.mdpi.com/article/10.3390/curroncol28060436/s1, Study protocol, Patient’s survey, Table S1 (Radiotherapy schedules and side effects), TableS2 (Endocrine therapy type, compliance and side effects).
