# Supplementary material for: Experiences and Perceptions of Older Adults with Lower-Risk Hormone Receptor-Positive Breast Cancer about Adjuvant Radiotherapy and Endocrine Therapy: A Patient Survey
Source: Curr Oncol. 2021 Dec 8;28(6):5215–26. doi: 10.3390/curroncol28060436 (PMC8700141; doi:10.3390/curroncol28060436)
Supplement: Supplementary file 1 [file curroncol-28-00436-s001.zip › curroncol-1465063-supplementary.pdf]

Oct 20, 2020

SUPPLEMENTAL MATERIAL

S1: Study protocol

First, do no harm. Evaluating the harms and benefits of radiotherapy and endocrine therapy in patients ≥70 years of age with low risk breast cancer. (REaCT-70 Survey)

STUDY PROTOCOL

Primary investigators: Dr. Marie-France Savard, Dr. Lynn Chang and Dr. Angel Arnaout

BACKGROUND:

Current guidelines recommend adjuvant radiotherapy and endocrine therapies for estrogen receptor (ER)-positive early stage breast cancer[1]. In a subset of the patients, the risks and side effects of taking radiotherapy and/or endocrine therapies may outweigh the benefits. This is a particular concern for elderly patients with a low risk breast cancer and comorbid medical conditions[2].

Clinical factors such as low-intermediate grade, tumor size 2 cm, and older age has been used successfully in a few studies to identify patients with a low risk of recurrence[3–7]. For instance, in the CALGB 9343 trial, despite the omission of adjuvant radiotherapy, the local and regional recurrence rates remains below 5% at 5 years and below 10% at 10 years without significant differences in time to mastectomy or , distant metastasis or breast cancer specific survival, in women ≥70 years with T1N0 breast cancer treated with adjuvant tamoxifen[4]. Furthermore, some studies suggest the existence of a less aggressive breast cancer biology in most patients ≥70 years of age[8].

Even though 30% of early stage breast cancers are diagnosed in patients ≥70 years , the elderly are often underrepresented in large prospective clinical trials evaluating the benefits of adjuvant therapies[2,9]. Acknowledging this underrepresented group, the FDA recently published a guidance for industry, named *Inclusions of Older Adults in Cancer Clinical Trials,* to assist and encourage stakeholders in the adequate representation of older adults in cancer clinical trials[10].

The acceptable benefit-risk profile of adjuvant radiotherapy and endocrine therapy remains to be defined for elderly patients with low risk breast cancer. We will conduct a survey among oncologists (i.e. medical oncologists, radiation oncologists, surgical oncologists and general practitioners in oncology/radiation) and older patients with a low risk breast cancer to address this issue and to determine the best adjuvant treatment de-escalation strategies. The information obtained from these surveys will help us to develop a pragmatic clinical trial that evaluates the risks and benefits of adjuvant radiotherapy and endocrine therapies in older patients with low risk breast cancer. The answers of the respondents will help us to align our research objectives with their priorities and integrate their voice into our research projects. We hope to use these responses to make future clinical studies more clinically relevant, patient-centered and practice-changing.

OBJECTIVES:

To evaluate physician and patient perceptions regarding:

1) The current practices involving adjuvant endocrine and radiotherapy in elderly patients with an ER-positive low risk breast cancer;
2) The side effects and impact on quality of life of adjuvant radiotherapy and endocrine therapy in elderly patients with an ER-positive low risk breast cancer;
3) The most appropriate de-escalating design and endpoints for a study evaluating risk and benefits of radiotherapy and endocrine therapy in elderly patients with an ER-positive low risk breast cancer

4) The importance of a future study evaluating risk and benefits of radiotherapy and endocrine therapy in elderly patients with an ER-positive low risk breast cancer.

METHODS:

Patient Survey

The survey will be explained to prospective participants during their clinic visit by a member of their circle of care (e.g. medical oncologists, radiation oncologists, surgical oncologists, general practitioners in oncology/radiation and nursing staff) and if interested, the patient will be given a paper copy of the survey and the information sheet. If the patient wishes for the survey to be emailed to them (and if permitted by the institution), then the study research assistant will send it from a secure hospital server. Patients will receive an email with the link to the survey on Microsoft Forms (on the Ottawa Hospital SharePoint site) which can be completed online. Or they can request to receive the questionnaire by email as a Word file or PDF file and can highlight their answers and email the completed survey back to the study staff. Surveys sent by email will be printed in hard copy (with no identifiers) and filed with the other paper copies. The survey can be completed at any time the patient wishes. The survey is anonymousand does not collect any personal identifying information. The questionnaire is anticipated to take 5-10 minutes to complete. Patients from three sites in Ontario will be participating.

Inclusion criteria

1) Patients with an ER-positive early stage (node negative) breast cancer treated with a lumpectomy or mastectomy;
2) Patients who were offered radiotherapy AND endocrine therapy, such as tamoxifen, anastrozole (*Arimidex*), letrozole (*Femara*) or exemestane (*Aromasin*);
3) Patients who are ≥70 years of age;
4) Able to provide verbal consent;
5) Willing and able to complete a survey in English.

Physician Survey

Physicians involved in the treatment of patients with ER-positive early stage (node-negative) breast cancer (e.g. medical oncologists, radiation oncologists, surgical oncologists and general practitioners in oncology/radiation) will be approached to participate in the physician survey. The Ottawa site has a collection of publicly available physicians email addresses that have been used in previous surveys of this type. The online survey will be run using Microsoft Forms on the Ottawa Hospital SharePoint site. Physicians will be emailed an information sheet and a link to the survey designed by the study team, which is anticipated to take 5-10 minutes to complete. The survey is completed anonymously (no personal identifiers will be collected).

Inclusion criteria

1) Physicians involved in the adjuvant treatment with radiotherapy and/or endocrine therapy of patients with an ER-positive early stage (node-negative) breast cancer, such as medical oncologists, radiation oncologists, surgical oncologists and general practitioners in oncology/radiation.
2) Willing and able to complete a survey in English.

STUDY ENDPOINTS:

Indicators physician and patient perceptions regarding:

Oct 20, 2020

1) The current practices involving adjuvant endocrine and radiotherapy in elderly patients with an ER-positive low risk breast cancer;
2) The side effects and impact on quality of life of adjuvant radiotherapy and endocrine therapy in elderly patients with an ER-positive low risk breast cancer;
3) The most appropriate de-escalating design and endpoints for a study evaluating risk and benefits of radiotherapy and endocrine therapy in elderly patients with an ER-positive low risk breast cancer;
4) The importance of a future study evaluating risk and benefits of radiotherapy and endocrine therapy in elderly patients with an ER-positive low risk breast cancer.

TIMELINE:

- *REB Submission*: April 2020
- *Data Collection: June-August 2020*
- *Statistical Analysis: September 2020*
- *Dissemination of Results: Fall 2020*

RECRUITMENT:

These surveys are expected to recruit 200 patients and 50 physicians. Participants will be asked to complete the survey in English.

DATA COLLECTION AND ANALYSIS:

Data collected in this study will include paper copies of the survey completed by patients, which will be secured in a locked filing cabinet at each local institution. Physicians willing to participate will be provided with the opportunity to complete the questionnaire online. All responses will be anonymous, and data will be stored with a platform that provides security, with password-limited access provided to the study team only. Collected data will be used to generate a database that will be managed using spreadsheet software (e.g. Excel) and digitally saved in a TOH encrypted server. Data analysis will generate current practice patterns in Ontario and also obtain the views of physicians and patients on acceptable benefit-risk profiles for adjuvant radiation and endocrine therapies as well as the best adjuvant treatment de-escalation strategy for elderly patients with low risk breast cancer. It will be presented descriptively.

FUNDING:

All costs associated with this study will be covered by internal funds, with no industry or pharmaceutical funding involved.

REFERENCES:

1. National Comprehensive Cancer Network. NCCN Clinical Practice Guidelines in Oncology: Breast Cancer, Version 3.2020. (2020). Available at: https://www.nccn.org/professionals/physician_gls/pdf/breast.pdf.
2. Lee, S. & Seo, J. H. Current Strategies of Endocrine Therapy in Elderly Patients with Breast Cancer. *Biomed Res. Int.* **2018**, 1–12 (2018).
3. Fisher, B. *et al.* Tamoxifen, Radiation Therapy, or Both for Prevention of Ipsilateral Breast Tumor Recurrence After Lumpectomy in Women With Invasive Breast Cancers of One Centimeter or Less. *J. Clin. Oncol.* **20**, 4141–4149 (2002).
4. Hughes, K. S. *et al.* Lumpectomy Plus Tamoxifen With or Without Irradiation in Women Age 70 Years or Older With Early Breast Cancer: Long-Term Follow-Up of CALGB 9343. *J. Clin. Oncol.* **31**, 2382–2387 (2013).

5. Sparano, J. A. *et al.* Clinical and Genomic Risk to Guide the Use of Adjuvant Therapy for BreastCancer. *N. Engl. J. Med.* **380**, 2395–2405 (2019).
6. Kunkler, I. H., Williams, L. J., Jack, W. J. L., Cameron, D. A. & Dixon, J. M. Breast-conservingsurgery with or without irradiation in women aged 65 years or older with early breast cancer (PRIME II): a randomised controlled trial. *Lancet Oncol.* **16**, 266–273 (2015).
7. Fyles, A. W. *et al.* Tamoxifen with or without Breast Irradiation in Women 50 Years of Age orOlder with Early Breast Cancer. *N. Engl. J. Med.* **351**, 963–970 (2004).
8. Downs-Canner, S. M. *et al.* Nodal positivity decreases with age in women with early-stage,
hormone receptor–positive breast cancer. *Cancer* **126**, 1193–1201 (2020).
9. DeSantis, C. E. *et al.* Breast cancer statistics, 2015: Convergence of incidence rates betweenblack and white women. *CA. Cancer J. Clin.* **66**, 31–42 (2016).
10. FDA. Inclusion of Older Adults in Cancer Clinical Trials Draft Guidance for Industry. *FDA-2019-D-5572* (2020). Available at: https://www.fda.gov/regulatory-information/search-fda- guidance-documents/inclusion-older-adults-cancer-clinical-trials.

June 21, 2021

S2: Patient's survey

Survey Title: First, do no harm. Evaluating the harms and benefits of radiotherapy and endocrine therapy in patients ≥70 years of age with low risk breast cancer. (REaCT-70 Survey)

A Survey for Patients

The purpose of this survey is to learn from early stage breast cancer patients who were offered **radiation therapy and hormonal therapy**, such as tamoxifen, anastrozole (Arimidex), letrozole (Femara) or exemestane (Aromasin). Completion of the survey implies your consent to participate in this survey.

We are trying to learn more about:

- o Your experience and perspectives on these treatments
- o How these treatments affect your well-being and lifestyle

1. How old were you when you were diagnosed with breast cancer? _________________

IF YOU WERE LESS THAN 70 YEARS OLD WHEN YOU WERE DIAGNOSED WITH BREAST CANCER, THEN DO NOT COMPLETE THE REST OF THE SURVEY

2. Was your cancer removed by either a mastectomy or a lumpectomy (sometimes called a partial mastectomy)?

a) Yes
b) No

IF YOU ANSWERED NO, THEN DO NOT COMPLETE THE REST OF THE SURVEY

3. Did your cancer doctors talk to you about both radiation therapy AND hormonal therapy, such as tamoxifen, anastrozole (Arimidex), letrozole (Femara) or exemestane (Aromasin)?

a) Yes
b) No

IF YOU ANSWERED NO, THEN DO NOT COMPLETE THE REST OF THE SURVEY

4. With respect to radiotherapy, please select the situation that applies to you:

a) I chose not to have radiotherapy
b) My radiotherapy will happen in the future
c) My radiotherapy is ongoing and not finished yet
d) I finished radiotherapy less than 3 months ago
e) I finished radiotherapy between 3 and 6 months ago
f) I finished radiotherapy between 6 and 12 months ago
g) I finished radiotherapy more than 12 months ago
h) Other – please say

5. With respect to hormone therapy (sometimes called endocrine therapy), please select the situation that applies to you:

a) I chose not to have hormonal therapy
b) I will take hormonal therapy in the future
c) I started taking hormonal therapy less than 3 months ago
d) I started taking hormonal therapy 3 to 6 months ago
e) I started taking hormonal therapy 6 to 12 months ago
f) I started taking hormonal therapy more than 12 months ago
g) I completed 5 years of hormonal therapy
h) Other – please say

6. How would you rate your health at the present time?

a) Excellent
b) Good
c) Fair
d) Poor
e) Bad

7. Approximately how many different prescribed medications do you take every day, excluding hormonal therapy medication (for example medication for high blood pressure)? _____________

8. Which of these conditions have you had or currently have? Please choose all that apply.

a) Diabetes or high blood sugars
b) Hypertension or high blood pressure
c) High cholesterol or dyslipidemia or lipids
d) Heart disease (ex: previous heart attack, previous stent, previous coronary bypass surgery, heart failure)
e) Previous stroke (or TIA/mini-stroke)
f) Kidney problems
g) Liver problems
h) Memory problems
i) Lung problems (ex: COPD, asthma)
j) Stomach ulcers
k) Mobility problems (ex: use of a cane, walker or a wheelchair)
l) Cancers other than breast cancer
m) Previous blood clots in your legs or lungs
n) Other, please specify: ____

We are planning a research study on the benefits and risks of radiotherapy and hormonal therapy in patients 70 years or older with an early stage breast cancer.

We would like to understand what you believe are acceptable benefits and risks of these treatments.

The next few questions are about radiotherapy

9. What do you think the radiotherapy does? Choose all that apply.

a) Helps to reduce the chances of cancer returning in the same breast
b) Helps to reduce the chances of cancer developing in the other breast
c) Helps to reduce the chances of cancer coming back in other organs, such as bone, lungs and liver
d) Helps to make me live longer
e) Helps to improve my quality of life
f) Could cause side effects without helping me

g) Don’t know, but I was told that it was important for me to take
h) Other, please specify: ____

10. What are your MAIN concerns about RADIOTHERAPY that might prevent you from taking the treatment? Choose all that apply.

a) Possible side effects
b) Negative impact on quality of life
c) Risk of not being able to do my daily activities
d) Chance of it not stopping the cancer coming back in my breast
e) Duration of the treatment
f) Difficulty getting from home to the hospital for the radiotherapy treatments
g) No significant concerns
h) Others: ____

11. How comfortable would you be if your doctor, based on your health and low risk cancer status, did NOT offer you RADIATION therapy?

a) Very comfortable
b) Comfortable
c) Somewhat comfortable
d) Somewhat uncomfortable
e) Uncomfortable
f) Very uncomfortable

For the next question, we will ask you about your willingness to receive radiotherapy based on the chances of your cancer coming back in your breast.

> For example, if the chance of the cancer coming back in your breast without radiotherapy is say 20% (1 in 5), I would only take radiotherapy if the chance is reduced by at least 5%, meaning that if I take the treatment there will be a 15% chance or less that the cancer returns instead of 20%.

12. I would ONLY take the radiotherapy therapy if, 5 years after being diagnosed, it reduces the chances of my cancer coming back in my breast by at least:

a) 1%
b) 5%
c) 10%
d) 15%
e) 20%
f) 30%
g) 50%

h) Reducing the chances of cancer coming back in my breast 5 years after my diagnosis is not important to me
i) I don't care how much it reduces the chance of it coming back, I will take any possible benefit

The next few questions are about hormone therapy

13. What do you think hormonal therapy does? Choose all that apply.

a) Helps to reduce the chances of cancer returning in the same breast
b) Helps to reduce the chances of cancer developing in the other breast
c) Helps to reduce the chances of cancer coming back in other organs, such as bone, lungs and liver
d) Helps to make me live longer
e) Helps to improve my quality of life
f) Could cause side effects without helping
g) I don't know, but I was told that it was important for me to take
h) Other, please specify: ____

14. What are your MAIN concerns about HORMONAL therapy that might prevent you from taking the treatment? Choose all that apply.

a) Possible side effects
b) Negative impact on quality of life
c) Risk of not being able to do my daily activities
d) Chance of the treatment not working
e) Duration of the treatment
f) Lack of benefit of hormonal therapy
g) No significant concerns
h) Other: ______________________________

15. How comfortable would you be if your doctor, based on your health and low risk cancer status, did NOT offer you HORMONAL therapy?

a) Very comfortable
b) Comfortable
c) Somewhat comfortable
d) Somewhat uncomfortable

e) Uncomfortable
f) Very uncomfortable

For the following 2 questions, we will ask you about your willingness to receive treatment based on the chances of your cancer coming back.

> For example, if the chance of the cancer coming is 20%, I would only take hormonal therapy if the chance is reduced by at least 5%, meaning that if I take the treatment there will be a 15% chance or less that the cancer returns instead of 20%.

16. I would ONLY take the hormonal therapy if, 5 years after being diagnosed, it reduces the chances of cancer coming back in my breast by at least:

a) 1%
b) 5%
c) 10%
d) 15%
e) 20%
f) 30%
g) 50%
h) Reducing the chances of cancer coming back in my breast 5 years after my diagnosis is not important to me
i) I don't care how much it reduces the chance of it coming back, I will take any possible benefit

17. I would ONLY take the hormonal therapy if, 5 years after my diagnosis, it reduces the chances of the breast cancer coming back elsewhere in my body by at least:

a) 1%
b) 5%
c) 10%
d) 15%
e) 20%
f) 30%
g) 50%
h) Reducing the chances of my breast cancer coming back elsewhere in my body 5 years after my diagnosis is not important to me
i) I don't care how much it reduces the chance of it coming back, I will take any possible benefit

18. I would ONLY take hormonal therapy if it increases my chances of being alive 5 years after my diagnosis by at least:

a) 1%

b) 2%
c) 5%
d) 10%
e) 20%
f) 30%
g) 50% or more
h) Increasing the chances of being alive 5 years after my diagnosis is not important to me
i) I don't care how much it increases my chances of being alive, I will take any possible benefit

We are planning to do a research study in patients of 70 years of age or older with low risk breast cancer. The goal is to evaluate the risks and benefits of hormonal therapy in this patient population. In this research trial, a computer, not a doctor, decides if a patient receives hormonal therapy or not. The decision is random and decided by chance alone.

Based on the information provided and/or on your experience from receiving hormonal therapy so far:

19. If your doctor told you that your breast cancer is low risk, how comfortable would you be participating in a research study where some of the patients will be receiving hormonal therapy and some will not?

a) Very comfortable
b) Comfortable
c) Unsure
d) Not comfortable
e) I am not interested in any kind of research study

Do you have any comments to add?

____
____
____

20. Did you receive:
a) Radiotherapy
b) Hormonal therapy

c) Both radiotherapy and hormonal therapy

IF YOU HAVE RECEIVED OR ARE RECEIVING RADIOTHERAPY, PLEASE ANSWER QUESTIONS 21 to 24

21. Duration and schedule of your radiotherapy:

a) 5 days per week for 3weeks
b) Once a week for 5 weeks
c) Biweekly (twice a week) for 2 and a half weeks
d) If unsure, please ask your treatment staff
e) Other – please say

22. Did you experience any of the following problems during radiation? Choose all that apply:

a) Skin redness
b) Peeling of skin
c) Breast swelling
d) Breast pain
e) Breast deformity
f) Worsening appearance of breast
g) Chest wall pain
h) Lymphedema (swelling of the arms)
i) Shoulder stiffness or arm mobility issues
j) Fatigue
k) Shortness of breath
l) Cough
m) Heart problems
n) Other, please specify

23. Did you experience any of the following problems 3 months or more, after the end of radiotherapy? Choose all that apply.

a) Skin redness
b) Peeling of skin

c) Breast swelling
d) Breast pain
e) Breast deformity
f) Worsening appearance of breast
g) Chest wall pain
h) Lymphedema (swelling of the arms)
i) Shoulder stiffness or arm mobility issues
j) Fatigue
k) Shortness of breath
l) Cough
m) Heart problems
n) Other, please specify

24. How did radiotherapy affect you?

a) No impact on my lifestyle or quality of life.
b) Minimal impact on lifestyle or quality of life.
c) Moderate impact on lifestyle or quality of life.
d) Major impact on lifestyle or quality of life, but resolved within 3 months
e) Major impact on lifestyle or quality of life, for more than 3 months
f) Major impact on lifestyle or quality of life, ongoing.

IF YOU HAVE RECEIVED OR ARE RECEIVING HORMONAL THERAPY, PLEASE ANSWER QUESTIONS 25 to 31

25. What type of hormonal therapy are you taking, or have you taken in the past? Choose all that apply.

a) Tamoxifen
b) Anastrozole (arimidex)
c) Exemestane (aromasin)
d) Letrozole (femara)
e) I have taken more than one type of hormonal therapy
f) Other, please specify: ____________________________________

26. If you answered e, I have taken more than one type of hormonal therapy, please give details:

____

27. Are you taking hormonal therapy as prescribed by your doctor?

a) Yes
b) Yes, but I forget or choose not to take one of the doses per week
c) Yes, but I forget or choose not to take up to half of the doses per week
d) Yes, but I forget or choose not to take more than half of the doses per week
e) I don’t take any of the prescribed hormonal therapy

28. If you answered b, c, d or e in the previous question, did you inform your doctor?

a) Yes
b) No
c) If not, why not? ____

29. In the year BEFORE you started receiving hormonal therapy, did you have any of the following problems? Choose all that apply.

a) Hot flashes
b) Night sweats
c) Joint pain
d) Muscle pain
e) Bone pain
f) Fracture
g) Vaginal bleeding
h) Blood clots
i) Stroke
j) Heart problems

k) Fatigue
l) Depression or anxiety
m) Headache
n) Nausea or vomiting
o) Cognitive impairment, memory problems
p) Unsure

30. WHILE receiving the hormonal therapy, did you have any of the following problems? Choose all that apply.

a) Hot flashes
b) Night sweats
c) Joint pain
d) Muscle pain
e) Bone pain
f) Fracture
g) Vaginal bleeding
h) Blood clots
i) Strokes
j) Heart problems
k) Fatigue
l) Depression or anxiety
m) Headache
n) Nausea or vomiting
o) Cognitive impairment, memory problems
p) Unsure

31. How did taking your hormonal therapy affect your life?

a) No impact on my lifestyle or quality of life.
b) Minimal impact on lifestyle or quality of life.
c) Moderate impact on lifestyle or quality of life.
d) Major impact on lifestyle or quality of life.

IF YOU HAVE RECEIVED BOTH RADIOTHERAPY AND HORMONAL THERAPY, PLEASE ANSWER QUESTIONS 32 to 34

32. Which treatment affected your quality of life the most?

a) Radiotherapy
b) Hormonal therapy
c) They both affected my quality of life equally
d) Neither affected my quality of life

33. If you had to choose between radiation therapy or hormonal therapy, which one would you rather have?

a) Hormonal therapy
b) Radiation therapy

34. Why would you choose this? ____

Thank you for participating in this survey. You are helping us to improve the care of our patients!

June 21, 2021

S3: Additional results

Table S1. Radiotherapy schedules and side effects

	N	N (%)
Duration and schedule	85	
5 days per week for 3 weeks		61 (72)
Once a week for 5 weeks		4 (5)
Biweekly (twice a week) for 2.5 weeks		2 (2)
Unsure		5 (6)
Other		13 (15)
Side effects reported during RT*	82	
Skin redness		55 (67)
Peeling of the skin		17 (21)
Breast swelling		13 (16)
Breast pain		16 (20)
Breast deformity		5 (6)
Worsening appearance of the breast		8 (10)
Chest wall pain		8 (10)
Lymphedema		1 (1)
Shoulder stiffness or arm mobility issue		9 (11)
Fatigue		40 (49)
Shortness of breath		4 (5)
Cough		3 (4)
Heart problems		0 (0)
Other, please specify		7 (9)
Side effects reported 3 months or more*	75	
Skin redness		11 (15)
Peeling of the skin		6 (8)
Breast swelling		8 (11)
Breast pain		13 (17)
Breast deformity		4 (5)
Worsening appearance of the breast		2 (3)
Chest wall pain		4 (5)
Lymphedema		3 (4)
Shoulder stiffness or arm mobility issue		10 (13)
Fatigue		25 (33)
Shortness of breath		3 (4)
Cough		2 (3)
Heart problems		0 (0)
Other, please specify		12 (16)

* participants were able to choose more than one answers

Table S2. Endocrine therapy type, compliance and side effects

	N	N (%)
Type of endocrine therapy*	80	
Tamoxifen		33 (40)
Anastrozole		33 (40)
Exemestane		0 (0)
Letrozole		19 (24)
More than one type		5 (6)
Compliance	80	
Take it as prescribed		66 (83)
Omit 1 dose / week		1 (1)
Omit up to half dose / week		1 (1)
Omit more than half dose / week		0
Don’t take it		12 (15)
Physician notified about non compliance	11	
Yes		9 (82)

No		1 (9)
Reported problems prior to start endocrine therapy*	80	
Hot flashes		8 (10)
Night sweats		4 (5)
Joint pain		31 (38)
Muscle pain		16 (20)
Bone pain		8 (10)
Fracture		5 (6)
Vaginal bleeding		0 (0)
Blood clots		2 (3)
Stroke		2 (3)
Heart problems		1 (1)
Fatigue		16 (20)
Depression or anxiety		8 (10)
Headache		5 (6)
Nausea or vomiting		0 (0)
Cognitive impairment, memory problems		2 (3)
Unsure		5 (6)
Other		12 (15)
Reported problems while receiving endocrine therapy*	80	
Hot flashes		36 (45)
Night sweats		23 (29)
Joint pain		36 (45)
Muscle pain		24 (30)
Bone pain		15 (19)
Fracture		0 (0)
Vaginal bleeding		1 (1)
Blood clots		0 (0)
Stroke		0 (0)
Heart problems		1 (1)
Fatigue		33 (41)
Depression or anxiety		10 (13)
Headache		7 (9)
Nausea or vomiting		3 (4)
Cognitive impairment, memory problems		7 (9)
Unsure		2 (3)
Other		10 (13)

*participants were able to choose more than one answers
